# Supplementary material for: Co-Amorphization, Dissolution, and Stability of Quench-Cooled Drug–Drug Coamorphous Supersaturating Delivery Systems with RT-Unstable Amorphous Components
Source: Pharmaceutics. 2024 Nov 21;16(12):1488. doi: 10.3390/pharmaceutics16121488 (PMC11677066; doi:10.3390/pharmaceutics16121488)
Supplement: Supplementary file 1 [file pharmaceutics-16-01488-s001.zip › pharmaceutics-3296770-supplementary.pdf]

# Supplement document

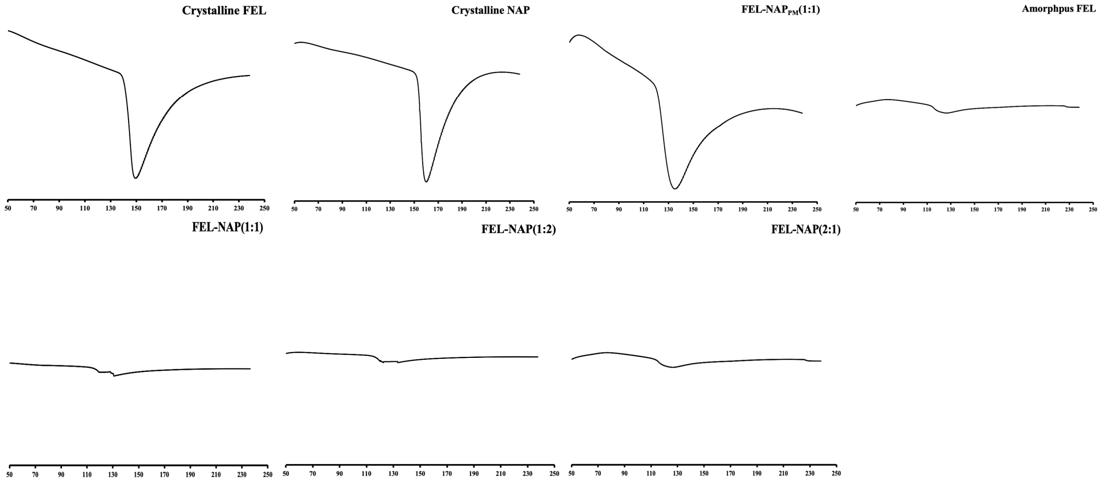


**Figure S1.** DSC thermograms of all samples, including crystalline FEL, crystalline NAP, FEL-NAP$_{PM}$ (1:1), amorphous FEL, FEL-NAP (1:1), FEL-NAP (1:2) and FEL-NAP (2:1).

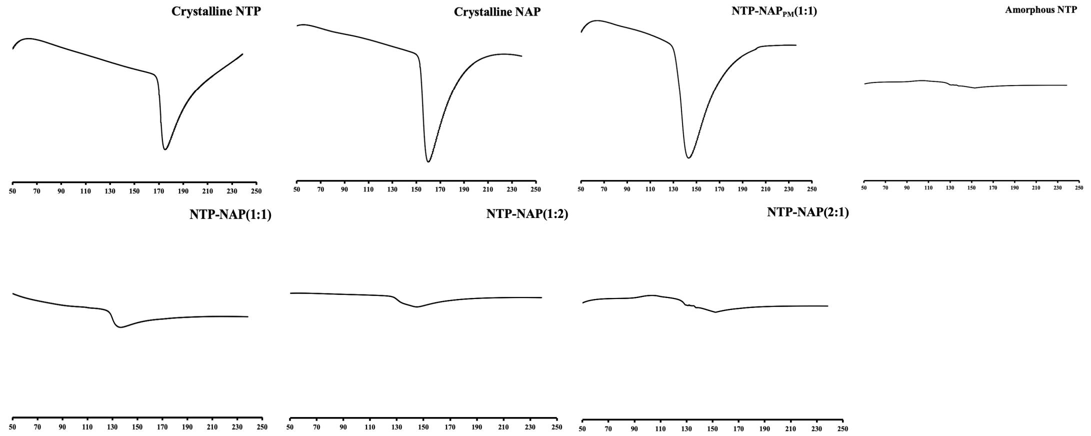


**Figure S2.** DSC thermograms of all samples including crystalline NTP, crystalline NAP, NTP-NAPPM (1:1), amorphous NTP, NTP-NAP (1:1), NTP-NAP (1:2) and NTP-NAP (2:1).

**Table S1** Dissolution rates of FEL-related samples including crystalline FEL, amorphous FEL, FEL-NAP$_{PM}$ and FEL-NAP (1:1, 1:2, and 2:1) by HPLC assay (mean ± SD, n=3). The crystalline FEL with amorphous FEL, FEL-NAP$_{PM}$ and FEL-NAP (1:1, 1:2, and 2:1) of statistical analysis were performed via unpaired student's t-test (* represents the $p$ < 0.05, ** represents the $p$ < 0.01 and *** represents the $p$ < 0.001.).

| Samples | Dissolution rates(μg/(mL·min) | | | | | |
|---|---|---|---|---|---|---|
| | 5 | 10 | 15 | 30 | 45 | 60 |
| Crystalline FEL | 0.25±0.089 | 0.16±0.020 | 0.16±0.019 | 0.090±0.019 | 0.066±0.010 | 0.062±0.002 |
| Amorphous FEL | 0.32±0.049* | 0.46±0.012** | 0.51±0.065** | 0.41±0.007*** | 0.42±0.011*** | 0.37±0.009*** |

| | | | | | | |
|---|---|---|---|---|---|---|
| FEL-NAP$_{PM}$ | 0.37±0.038$^{*}$ | 0.28±0.093$^{*}$ | 0.30±0.023$^{**}$ | 0.24±0.015$^{***}$ | 0.25±0.005$^{***}$ | 0.23±0.005$^{***}$ |
| FEL-NAP (1:1) | 0.49±0.014$^{**}$ | 1.02±0.070$^{***}$ | 1.09±0.084$^{***}$ | 0.78±0.008$^{***}$ | 0.57±0.013$^{***}$ | 0.47±0.006$^{***}$ |
| FEL-NAP (1:2) | 0.45±0.085$^{**}$ | 1.64±0.056$^{***}$ | 1.51±0.090$^{***}$ | 1.07±0.009$^{***}$ | 0.78±0.004$^{***}$ | 0.64±0.008$^{***}$ |
| FEL-NAP (2:1) | 0.38±0.057$^{*}$ | 1.39±0.050$^{***}$ | 1.33±0.021$^{***}$ | 0.94±0.035$^{***}$ | 0.70±0.005$^{***}$ | 0.57±0.004$^{***}$ |

**Table S2** Dissolution rates of NAP-related samples including crystalline NAP, FEL-NAP$_{PM}$ and FEL-NAP (1:1, 1:2, and 2:1) by HPLC assay (mean ± SD, n=3). The crystalline NAP with FEL-NAP$_{PM}$ and FEL-NAP (1:1, 1:2, and 2:1) of statistical analysis were performed via unpaired student's t-test (* represents the $p < 0.05$).

| Samples | Dissolution rates(μg/(mL·min) | | | | | |
|---|---|---|---|---|---|---|
| | 5 | 10 | 15 | 30 | 45 | 60 |
| Crystalline NAP | 2.09±0.29 | 1.12±0.47 | 0.79±0.29 | 0.44±0.13 | 0.33±0.022 | 0.27±0.039 |
| FEL-NAP$_{PM}$ | 2.16±0.33 | 1.18±0.23 | 0.82±0.16 | 0.49±0.036 | 0.37±0.042 | 0.30±0.021 |
| FEL-NAP (1:1) | 2.71±0.38 | 1.51±0.35 | 1.12±0.36$^{*}$ | 0.69±0.035$^{*}$ | 0.54±0.034$^{*}$ | 0.42±0.012$^{*}$ |
| FEL-NAP (1:2) | 2.85±0.45 | 1.60±0.23 | 1.20±0.23$^{*}$ | 0.75±0.049$^{*}$ | 0.57±0.046$^{*}$ | 0.46±0.079$^{*}$ |
| FEL-NAP (2:1) | 2.52±0.17 | 1.39±0.36 | 1.04±0.29$^{*}$ | 0.63±0.17$^{*}$ | 0.50±0.063$^{*}$ | 0.41±0.035$^{*}$ |

**Table S3** Dissolution rates of NTP-related samples including crystalline NTP, amorphous NTP, NTP-NAP$_{PM}$ and NTP-NAP (1:1, 1:2, and 2:1) by HPLC assay (mean ± SD, n=3). The crystalline NTP with amorphous NTP, NTP-NAP$_{PM}$ and NTP-NAP (1:1, 1:2, and 2:1) of statistical analysis were performed via unpaired student's t-test (* represents the $p < 0.05$, ** represents the $p < 0.01$ and *** represents the $p < 0.001$).

| Samples | Dissolution rates(μg/(mL·min)*$10^{-3}$) | | | | |
|---|---|---|---|---|---|
| | 10 | 15 | 30 | 45 | 60 |
| Crystalline NTP | 6.64±0.022 | 12.11±0.013 | 26.13±0.028 | 28.37±0.14 | 33.92±0.30 |
| Amorphous NTP | 73.92±0.032$^{***}$ | 114.16±0.12$^{***}$ | 109.58±0.062$^{***}$ | 103.88±0.20$^{***}$ | 93.33±0.093$^{***}$ |
| NTP-NAP$_{PM}$ | 20.87±0.038$^{***}$ | 35.10±0.15$^{**}$ | 41.65±0.090$^{**}$ | 47.86±0.33$^{**}$ | 48.61±0.21$^{*}$ |
| NTP-NAP (1:1) | 9.44±0.013$^{*}$ | 47.02±0.022$^{**}$ | 76.67±0.090$^{**}$ | 68.88±0.33$^{**}$ | 64.13±0.30$^{**}$ |
| NTP-NAP (1:2) | 34.95±0.027$^{***}$ | 76.33±0.043$^{***}$ | 98.48±0.051$^{***}$ | 86.03±0.19$^{***}$ | 78.03±0.14$^{**}$ |
| NTP-NAP (2:1) | 184.18±0.025$^{***}$ | 179.31±0.14$^{***}$ | 165.96±0.30$^{***}$ | 160.56±0.087$^{***}$ | 149.86±0.63$^{***}$ |

**Table S4** Dissolution rates of NAP-related samples including crystalline NAP NTP-NAP$_{PM}$ and NTP-NAP (1:1, 1:2, and 2:1) by HPLC assay (mean ± SD, n=3).

The crystalline NAP with NTP-$NAP_{PM}$ and NTP-NAP (1:1, 1:2, and 2:1) of statistical analysis were performed via unpaired student's t-test (** represents the $p < 0.01$ and *** represents the $p < 0.001$).

| Samples | Dissolution rates(μg/(mL·min)) | | | | | |
|---|---|---|---|---|---|---|
| | 5 | 10 | 15 | 30 | 45 | 60 |
| Crystalline NAP | 0.028±0.015 | 0.051±0.019 | 0.082±0.031 | 0.058±0.011 | 0.046±0.005 | 0.048±0.006 |
| NTP-$NAP_{PM}$ | $0.10±0.066^{***}$ | $0.097±0.008^{**}$ | $0.16±0.014^{**}$ | $0.11±0.054^{**}$ | $0.085±0.018^{**}$ | $0.095±0.062^{**}$ |
| NTP-NAP (1:1) | $0.28±0.012^{***}$ | $0.45±0.026^{***}$ | $0.48±0.11^{***}$ | $0.51±0.064^{***}$ | $0.55±0.077^{***}$ | $0.51±0.060^{***}$ |
| NTP-NAP (1:2) | $0.40±0.023^{***}$ | $0.57±0.033^{***}$ | $0.59±0.055^{***}$ | $0.67±0.075^{***}$ | $0.64±0.089^{***}$ | $0.60±0.041^{***}$ |
| NTP-NAP (2:1) | $0.23±0.088^{***}$ | $0.36±0.058^{***}$ | $0.35±0.034^{***}$ | $0.38±0.045^{***}$ | $0.43±0.028^{***}$ | $0.43±0.072^{***}$ |
